# A quantum walk simulation of extra dimensions with warped geometry

**DOI:** 10.1038/s41598-022-05673-2

**Published:** 2022-02-04

**Authors:** Andreu Anglés-Castillo, Armando Pérez

**Affiliations:** Departament de Fìsica Teórica & IFIC, Universitat de València-CSIC, 46100 Burjassot, València Spain

**Keywords:** Quantum information, Quantum mechanics, Quantum simulation

## Abstract

We investigate the properties of a quantum walk which can simulate the behavior of a spin 1/2 particle in a model with an ordinary spatial dimension, and one extra dimension with warped geometry between two branes. Such a setup constitutes a $1+1$ dimensional version of the Randall–Sundrum model, which plays an important role in high energy physics. In the continuum spacetime limit, the quantum walk reproduces the Dirac equation corresponding to the model, which allows to anticipate some of the properties that can be reproduced by the quantum walk. In particular, we observe that the probability distribution becomes, at large time steps, concentrated near the “low energy” brane, and can be approximated as the lowest eigenstate of the continuum Hamiltonian that is compatible with the symmetries of the model. In this way, we obtain a localization effect whose strength is controlled by a warp coefficient. In other words, here localization arises from the geometry of the model, at variance with the usual effect that is originated from random irregularities, as in Anderson localization. In summary, we establish an interesting correspondence between a high energy physics model and localization in quantum walks.

## Introduction

Quantum walks (QWs) constitute an interesting possibility for simulating physical phenomena from many fields. The discrete time version describes the motion of a spin 1/2 particle on a lattice. For instance, by simply incorporating suitable position-dependent phases on the unitary operator that implements the time evolution, one can mimic the effects of an external electromagnetic field^1–8^. In the continuum limit (when both the time step and the lattice spacing tend to zero), the Dirac equation in presence of such fields is recovered. In an analogous way, the motion of a Dirac particle in presence of a gravitational field can be simulated by an appropriate choice of the operator that drives the evolution, either on a rectangular or other types of lattices^3,9,10^. Other scenarios include vacuum or matter neutrino oscillations^11–13^, and one can even establish some connections to lattice field theories^14^.

There is also a different connection of QWs with quantum field theories, namely the possibility to explore some models which include extra dimensions, which are only manifested at very high energies. The possibility of extra dimensions of space was first suggested by Theodor Kaluza and Oscar Klein^15,16^ seeking an unified theory of electromagnetic and gravitational fields into a higher dimensional field, with one of the dimensions compactified. Experimental data from particle colliders restrict the compactification radius to such small scales that it becomes virtually impossible to explore these extra dimensions. Different ideas have been proposed to overcome this difficulty, for example the domain wall model introduced by Rubakov and Shaposhnikov^17^, in which the particle couples to an external scalar field. The motion of a spin 1/2 particle moving inside such a geometry was analyzed in Ref.^18^. In addition to recovering the corresponding Dirac equation in the continuum limit, the QW shows, at finite spacetime spacing, localization of the particle within the brane due to the coupling to the field.

Spatial localization is an important phenomenon in physics, which appears within the context of diffusion processes in lattices. It can arise from random noise on the lattice sites, giving rise to Anderson localization^19^ and causing a metal-insulator transition, but it can also be the consequence of the action of an external periodic potential (see e.g. Refs.^20–22^). Similarly, one obtains localization for the 1-dimensional QW when spatial disorder is included^23–25^, non-linear effects^26^, or by the use of a spatially periodic coin^27^. The results in Ref.^18^ show, however, that localization can also appear as a consequence of the interaction with a* smooth* external potential, instead of a random, or even periodic, perturbation.

In this paper, we investigate localization effects that arise within a different context, which is also inspired on high energy physics, and was originally proposed to address the *hierarchy problem* (the observed difference between the Higgs mass, and the Planck scale, in many orders of magnitude), and is commonly referred to as the Randall–Sundrum model^28^. This model assumes an extra dimension which extends between two *branes* (with a topology that will be discussed later). Here we consider a simplified version with one ordinary spatial dimension and one extra dimension, and define a QW that reproduces the dynamics of a spin 1/2 particle in the continuum spacetime limit.

Unlike the Rubakov and Shaposhnikov model, there is no coupling to an external scalar field. Instead, this model presents a warped geometry along the extra dimension. As we will show, this curvature is at the root of a localization effect of the QW towards the second (low energy) brane. The stationary states of the model in the continuum limit become concentrated close to the low energy brane for high values of the warp coefficient, which quantifies the strength of the localization. The localization of the QW can be analyzed by quantifying its overlap with these stationary states. This allows us to tailor the dynamics of the QW, showing a different behavior as the value of the warp coefficient is changed. In this way, we arrive at a QW model with a rich phenomenology, where some properties are inherited from the continuum field theoretic model. There is, in this sense, a mutual multidisciplinary benefit: one can design a QW which simulates an important high energy physics model. In exchange, the knowledge of the continuum properties is useful to understand, and to control, the dynamics of the QW in different regimes.

This paper is organized as follows. We first define the Randall–Sundrum model in $1+1$ spatial dimensions, along with its main properties. We pay special attention to the stationary states of the Hamiltonian, which play a crucial role in understanding the dynamics of the proposed QW. Next, we define a QW which allows to recover the dynamics of the Randall–Sundrum model for a spin 1/2 particle, and we study its phenomenology. Namely, we show that the distribution probability, as well as the expected value of the position along the extra dimension, approaches the lower brane at large time, and that this approaching proceeds more slowly for larger values of the warp coefficient, which turns out to be the main parameter in controlling the dynamics. We also analyze the entanglement entropy between spatial and internal degrees of freedom, exhibiting a complex behavior as a function of that parameter, which can be attributed to the different sharpness of the probability distribution. We finally conclude by collecting and discussing our main results.

## The model

### Orbifold $S^{1}/{\mathbb {Z}}_{2}$ and background geometry

**Figure 1:**
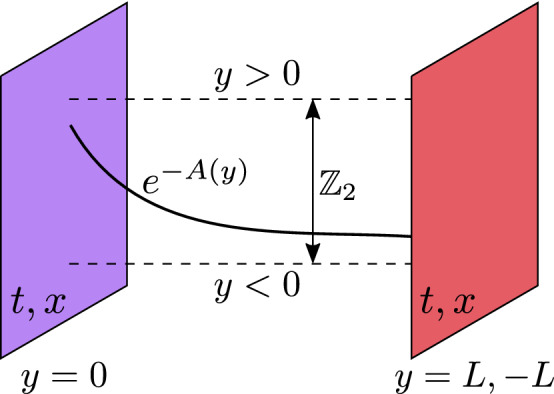
Schematic representation of the extra dimension in the Randall–Sundrum model.

As described in the “Introduction”, we consider the Randall–Sundrum model (RSM)^28^ with a single extra dimension *y*, together with a 2-dimensional ordinary spacetime, whose coordinates are denoted by $x^{\mu }=\{t,x\}$. The total spacetime possesses $D=3$ dimensions. The extra dimension *y* is compactified on a circle of radius *R*, and subject to a ${\mathbb {Z}}_{2}$ symmetry. These features are captured by the equivalences

1 $$\begin{aligned} S^{1}:\;y&\sim y+2\pi R~, \end{aligned}$$

2 $$\begin{aligned} {\mathbb {Z}}_{2}:\;y&\sim -y, \end{aligned}$$

which define the orbifold $S^{1}/{\mathbb {Z}}_{2}$ describing this extra dimension. Along the *y* dimension, the orbifold is a finite segment with two fixed points at $y=0$ and $y=\pi R\equiv L$. The RSM assumes that there is a $(D-1)$-brane of ordinary dimensions at each fixed point, see Fig. 1 for a sketch of the space configuration and the orbifold symmetries.

The matter fields are supposed to reside on the brane at $y=L$, which is referred to as the “visible brane”, while the brane at $y=0$ is the “hidden brane”. Both branes contribute to the bulk background geometry through their tensions, or vacuum energies, $T_{\mathrm {vis}}$ and $T_{\mathrm {hid}}$ respectively^28,29^. The total background action is

3 $$\begin{aligned} S=\int _{-L}^{L}dy\int dx^{\mu }\sqrt{|g|}\left( 2\alpha {\mathscr {R} }-\Lambda \right) +S_{\mathrm {vis}}+S_{\mathrm {hid}}, \end{aligned}$$

where the first term is the usual Einstein–Hilbert action of the total space, with $\Lambda$ the bulk cosmological constant, $\alpha$ a constant and |*g*| the absolute value of the metric determinant, while

4 $$\begin{aligned} S_{\mathrm {vis}}&=-\int dx^{\mu }\sqrt{|g_{\mathrm {vis}}|}T_{\mathrm {vis}}, \end{aligned}$$

5 $$\begin{aligned} S_{\mathrm {hid}}&=-\int dx^{\mu }\sqrt{|g_{\mathrm {hid}}|}T_{\mathrm {hid}}, \end{aligned}$$

are the action contributions of the branes tensions, with the induced metrics $g_{\mathrm {vis}}(x^{\mu })=g(x^{\mu },y=L)$ and $g_{\mathrm {hid}}(x^{\mu })=g(x^{\mu },y=0)$. To address the hierarchy problem, the following metric was proposed

6 $$\begin{aligned} ds^{2}=e^{-2A(y)}\eta _{\mu \nu }dx^{\mu }dx^{\nu }-dy^{2}, \end{aligned}$$

where $e^{-2A(y)}$ is a *warp factor*, a rapidly changing function along the additional dimension, and $\eta _{\mu \nu }$ is the Minkowski metric with signature $(+,-)$. The metric in Eq. (6) obeys Einstein’s equations that are obtained from the action (3): We refer the reader to the Supplementary Information for the standard computation particularized to this lower-dimensional spacetime. We also show that, as a consequence of these equations, the function in the exponent is given by

7 $$\begin{aligned} A(y)=k|y|, \end{aligned}$$

where *k* is the so called *warp coefficient*.

### Fermions in the Randall–Sundrum model

We now focus on the study of spin 1/2 fermions, whose evolution equation is the Dirac equation in curved spacetime

8 $$\begin{aligned} (i\gamma ^{a}e_{a}^{\mu }D_{\mu }-m)\Psi =0. \end{aligned}$$

The $\gamma ^{a}$ are the Dirac gamma matrices in a local rest frame, and the covariant derivative is

9 $$\begin{aligned} D_{\mu }=\partial _{\mu }-\frac{i}{4}\omega _{\mu }^{ab}\sigma _{ab}, \quad \text {with}\quad \sigma _{ab}=\frac{i}{2}[\gamma _{a},\gamma _{b}], \end{aligned}$$

where $\omega _{\mu }^{ab}$ is the spin connection. The *vierbeins* $e_{a}^{\mu }$ allow to express the Dirac matrices in a rest frame, that is, they perform a change of basis to a non-coordinate system in which the metric becomes the Minkowski metric

10 $$\begin{aligned} g_{\mu \nu }e_{a}^{\mu }e_{b}^{\nu }=\eta _{ab}. \end{aligned}$$

Equation (8) defines the vector current

11 $$\begin{aligned} j^{\mu }=\sqrt{|g|}e_{a}^{\mu }{\overline{\Psi }}\gamma _{a}\Psi , \end{aligned}$$

whose conservation $\partial _{\mu }j^{\mu }=0$ imposes the normalization condition

12 $$\begin{aligned} \int dx^{\mu }\sqrt{|g|}e_{0}^{0}\Psi ^{\dagger }\Psi =1. \end{aligned}$$

In the case of 2 spatial dimensions, the Dirac equation (8) can be reduced, after some algebra, to

13 $$\begin{aligned} i\gamma ^{a}\left[ e_{a}^{\mu }\partial _{\mu }\Psi +\frac{1}{2\sqrt{|g|}} \partial _{\mu }(e_{a}^{\mu }\sqrt{|g|})\Psi \right] -m\Psi =0, \end{aligned}$$

where the $\gamma _{a}$ matrices become Pauli matrices. A simple choice of the vierbein obeying relation (10) is

14 $$\begin{aligned} e_{0}=(e^{A(y)},0,0),\quad e_{1}=(0,e^{A(y)},0),\quad e_{2}=(0,0,1), \end{aligned}$$

which yields the following expression for the Dirac equation

15 $$\begin{aligned} i\partial _{t}\Psi =-i\gamma ^{0}\gamma ^{1}\partial _{x}\Psi -i\gamma ^{0} \gamma ^{2}\partial _{y}(e^{-A(y)}\Psi )+\gamma _{0}e^{-A(y)}m\Psi . \end{aligned}$$

This expression can be rewritten in Hamiltonian form as

16 $$\begin{aligned} i\partial _{t}\chi ={\mathscr {H}}\chi , \end{aligned}$$

with

17 $$\begin{aligned} {\mathscr {H}}=-\frac{i}{2}\{B^{x},\partial _{x}\} -\frac{i}{2}\{B^{y},\partial _{y}\}+\gamma _{0}e^{-A(y)}m, \end{aligned}$$

where the change of variable $\chi =e^{-A(y)/2}\Psi$ was performed, and we defined

18 $$\begin{aligned} B^{x}=\gamma ^{0}\gamma ^{1}~,\quad B^{y}=e^{-A(y)}\gamma ^{0}\gamma ^{2}. \end{aligned}$$

The symbol $\{\cdot ,\cdot \}$ represents the anticommutator of two operators. There is some freedom in the choice of the gamma matrices. For convenience, we choose

19 $$\begin{aligned} \gamma ^{0}=\sigma _{x},\quad \gamma ^{1}=i\sigma _{y}, \quad \gamma ^{2}=i\sigma _{z}. \end{aligned}$$

### Boundary conditions for fermionic fields

The periodic condition (1) simply implies that the fermionic fields need also to be periodic

20 $$\begin{aligned} \chi (x^{\mu },y+2L)=\chi (x^{\mu },y), \end{aligned}$$

but the ${\mathbb {Z}}_{2}$ needs a deeper consideration, since it has to leave the fermionic action invariant. We can write the fermionic action as

21 $$\begin{aligned} S_{F}=\int dx^{\mu }{\int _{-L}^{L} dy}{\overline{\chi }}(x^{\mu },y) \left( i\gamma ^{\mu }\partial _{\mu }+i\gamma ^{2}\partial _{y} e^{-A(y)}-e^{-A(y)}{{m}}\right) \chi (x^{\mu },y). \end{aligned}$$

which is extremized by the Dirac equation (15). Under ${\mathbb {Z}}_{2}$, the fermionic action becomes

22 $$\begin{aligned} S_{F}=\int dx^{\mu }{\int _{-L}^{L} dy}{\overline{\chi }}(x^{\mu }, -y)\left( i\gamma ^{\mu }\partial _{\mu }-i\gamma ^{2}\partial _{y} e^{-A(-y)}-e^{-A(-y)}m\right) \chi (x^{\mu },-y). \end{aligned}$$

The general boundary condition for a fermionic field under ${\mathbb {Z}}_{2}$ is given by $\chi (x^{\mu },-y)=T_{\chi }[{\mathbb {Z}}_{2}]\chi (x^{\mu },y)$^30,31^, where $T_{\chi }[{\mathbb {Z}}_{2}]$ is the matrix representation for the action of ${\mathbb {Z}}_{2}$. We rename it to $T_{\chi }[{\mathbb {Z}}_{2}]=M$ to alleviate the notation. We then need to find an operator *M* that keeps the action invariant. The action (22) is therefore transformed as

23 $$\begin{aligned} S_{F}=\int dx^{\mu }{\int _{-L}^{L}}dy{\overline{\chi }}(x^{\mu },y) \gamma ^{0}{M^{\dagger }}\gamma ^{0}\left( i\gamma ^{\mu }\partial _{\mu } -i\gamma ^{2}\partial _{y}e^{-A(y)}-e^{-A(y)}m\right) {M}\chi (x^{\mu },y), \end{aligned}$$

and establishes the following restrictions for *M* to keep the action (21) invariant,

24 $$\begin{aligned}&\gamma ^{0}{M^{\dagger }}\gamma ^{0}\gamma ^{\mu }{M}=\gamma ^{\mu }, \end{aligned}$$

25 $$\begin{aligned}&\gamma ^{0}{M^{\dagger }}\gamma ^{0}\gamma ^{2}{M}=-\gamma ^{2}, \end{aligned}$$

26 $$\begin{aligned}&\gamma ^{0}{M^{\dagger }}\gamma ^{0}{M}={\mathbb {I}}, \end{aligned}$$

where the first 2 conditions come from the kinetic terms of the action, and the last one arises from the mass term. There does not exist a solution for *M* that solves all conditions simultaneously, although $M=\eta \sigma _{z}$ is a solution for the first 2, with $\eta =\pm 1$ (since $M^{2}={\mathbb {I}}$). This means that a constant mass term is forbidden. In the following we restrict ourselves to the case where the “bulk mass” *m* vanishes. The action of the fermionic field is therefore

27 $$\begin{aligned} \begin{aligned} S_{F}&= \int dx^{\mu }{\int _{-L}^{L} dy}{\overline{\chi }}(x^{\mu },y) \left( i\gamma ^{\mu }\partial _{\mu }+i\gamma ^{2}\partial _{y}e^{-A(y)} \right) \chi (x^{\mu },y), \end{aligned} \end{aligned}$$

and the fermionic field has to obey the boundary condition

28 $$\begin{aligned} \chi (x^{\mu },-y)=\eta \sigma _{z}\chi (x^{\mu },y), \end{aligned}$$

with $$\eta =\pm 1$$.

**Figure 2:**
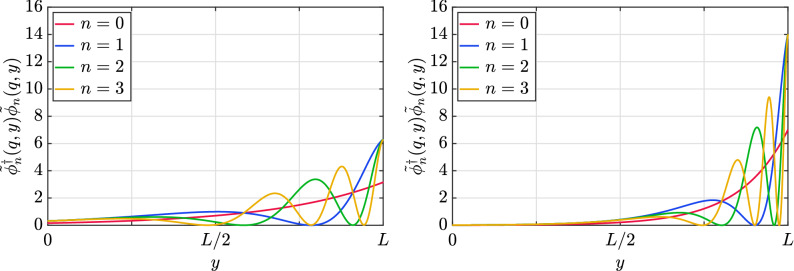
Plots of the probability distribution for the first four stationary states, with positive energy and a value of $q=10$, for $kL=3$ on the left and for $kL=7$ on the right.

### Stationary solutions

In this model, the Dirac field satisfies a complicated equation, Eq. (15), which is difficult to address even numerically. In order to obtain some insight, we first look for stationary solutions, which are defined as the eigenstates of the Hamiltonian. For $m=0$, and with our choice of the gamma matrices, the Hamiltonian takes the form

29 $$\begin{aligned} {\mathscr {H}}=-\sigma _{z}{\hat{p}}_{x}+\frac{\sigma _{y}}{2} \left( e^{-A(y)}{\hat{p}}_{y}+{\hat{p}}_{y}e^{-A(y)}\right) , \end{aligned}$$

where $p_{k}=-i\partial _{k}$ is the momentum operator along the *k* direction ($k=x,y$). The stationary states $\phi _{n}(x,y)$ corresponding to energy $E_{n}$ satisfy

30 $$\begin{aligned} {\mathscr {H}}\phi _{n}(x,y)=E_{n}\phi _{n}(x,y). \end{aligned}$$

It is convenient to introduce a Fourier transform on the ordinary dimension *x*:

31 $$\begin{aligned} {\tilde{\phi }}_{n}(q,y)=\int dxe^{-iqx}\phi _{n}(x,y), \end{aligned}$$

since the field is free to move along this direction. We found the energies

32 $$\begin{aligned} E_{n}=\pm \sqrt{q^{2}+\left( k\alpha _{n}\right) ^{2}}, \end{aligned}$$

where

33 $$\begin{aligned} \alpha _{n}=\frac{n\pi }{e^{kL}-1}~,~n=0,1,\dots \end{aligned}$$

The eigenfunctions associated with this spectrum that satisfy the boundary condition (28), for the particular case with $\eta =1$, are

34 $$\begin{aligned} {\tilde{\phi }}_{n}^{\uparrow }(q,y)&=\sqrt{\frac{2k}{e^{kL}-1}} \frac{E_{n}+q}{\sqrt{(E_{n}+q)^{2}+(k\alpha _{n})^{2}}} e^{\frac{k|y|}{2}}\cos \left[ \alpha _{n}\left( e^{k|y|}-1\right) \right] , \end{aligned}$$

35 $$\begin{aligned} {\tilde{\phi }}_{n}^{\downarrow }(q,y)&=\sqrt{\frac{2k}{e^{kL}-1}} \frac{k\alpha _{n}}{\sqrt{(E_{n}+q)^{2}+(k\alpha _{n})^{2}}} e^{\frac{k|y|}{2}}\sin \left[ \alpha _{n}\left( e^{k|y|}-1\right) \right] \mathrm {sign}(y), \end{aligned}$$

where the components of the spinor field are ${\tilde{\phi }}_{n}=({\tilde{\phi }}_{n}^{\uparrow },{\tilde{\phi }}_{n}^{\downarrow })^{T}$. The particular case $n=0$ only has an upper component, which is given by

36 $$\begin{aligned} {\tilde{\phi }}_{0}^{\uparrow }(q,y)=\sqrt{\frac{k}{e^{kL}-1}} e^{\frac{k|y|}{2}}\mathrm {sign}(E_{n}+q), \end{aligned}$$

and is undefined for energy and momentum with different sign. The procedure to obtain the eigenfunctions is detailed in the Supplementary Information, as well as the solution for $\eta =-1$. The probability distribution associated to these wavefunctions is concentrated around $y=L$ for high values of the warp coefficient *k*. We illustrate this behavior in Fig. 2, where we have plotted the probability density for the first modes with positive energy, and momentum $q=10$, for a value of the warp coefficient $kL=3$ and $kL=7$, respectively.

## A quantum walk for the Randall–Sundrum model

Once we have discussed the main properties of the RSM in the continuum spacetime, we focus on the main goal of our work, which consists in constructing a QW that is able to simulate the dynamics of a spin 1/2 particle subject to the geometric effects and symmetries of the model. To incorporate the metric, we adapt the scheme introduced in Ref.^9^, which allows to reproduce (in the continuum limit) a Dirac equation of the form Eq. (16).

The QW is defined on a 2-dimensional discrete grid with *x* and *y* axis, with discrete positions labeled by *r* and *s*, respectively. The grid points are equally spaced by $\varepsilon$, so that the spatial coordinates can be related to the grid points by $x=\varepsilon r$ and $y=\varepsilon s$. The Hilbert space that corresponds to these spatial degrees of freedom, ${\mathscr {H}}_{spatial}$ is spanned by the basis $\{|x=\varepsilon r,y=\varepsilon s\rangle \}/r,s\in {\mathbb {Z}}$. Time steps are labeled by $j\in {\mathbb {N}}$, and are also equally spaced by $\varepsilon$. The coin (or internal) space is a 2 dimensional Hilbert space ${\mathscr {H}}_{\mathrm {coin}}$, so that the total Hilbert space is ${\mathscr {H}}_{tot}={\mathscr {H}}_{spatial}\otimes {\mathscr {H}}_{\mathrm {coin}}$. At a given time step, the state of the walker will be represented by a two component spinor $|\chi _{j}\rangle \in {\mathscr {H}}_{tot}$. The one step evolution of the QW is given by

37 $$\begin{aligned} |\chi _{j+1}\rangle =U|\chi _{j}\rangle , \end{aligned}$$

where we made use of the general operator introduced in Ref.^9^. The structure of U consists on alternating displacement operators along each direction, together with unitary operators which are functions of some angle that is allowed to be spacetime dependent. Since the displacement operator and the position-dependent unitaries do not commute in general, a term containing the spatial derivative of those unitaries appears in the continuum limit, which is needed for a construction that takes the form of Eq. (17). The angles appearing in this general expression have to be chosen to reproduce the appropriate operators $B^{x}$ and $B^{y}$ given by Eq. (18) that correspond to the metric (or vierbein) of the model. Some of these angles are trivial in our case, so that one arrives to a simplified expression, given by (details are given in the Supplementary Information):

38 $$\begin{aligned} U=R^{-1}(y)\left[ \Theta (y)S_{y}({-}\varepsilon /2) \right] ^{2}R(y)S_{x}({-}\varepsilon ), \end{aligned}$$

where $S_{k}(\varepsilon )=\exp (-i\sigma _{z}p_{k}\varepsilon )$ are spin-dependent shift operators in the direction $\pm k$ (with $k=x,y$),

39 $$\begin{aligned} \Theta (y)=\begin{pmatrix}{-}c(y) &{} is(y)\\ -is(y) &{} c(y) \end{pmatrix}, \end{aligned}$$

with $$c(y)=e^{-A(y)}$$, $$s(y)=\sqrt{1-e^{-2A(y)}}$$, and

40 $$\begin{aligned} R(y)=\frac{1}{\sqrt{2}}\begin{pmatrix}f^{*}(y) &{} if(y)\\ -f^{*}(y) &{} if(y) \end{pmatrix}, \end{aligned}$$

where $f(y)=\sqrt{\frac{1+c(y)}{2}}+i\sqrt{\frac{1-c(y)}{2}}$. At each position (*r*, *s*) we introduce

41 $$\begin{aligned} \chi _{j,r,s}\equiv \langle x=\varepsilon r,y =\varepsilon s|\chi _{j}\rangle =\begin{pmatrix}\chi _{j,r,s}^{\uparrow }\\ \chi _{j,r,s}^{\downarrow } \end{pmatrix}, \end{aligned}$$

which represents the amplitude (given a component of the spin) for the particle to be localized at the position labeled by (*r*, *s*) and time step *j*. In this way, the time step defined by (37) can be recast as a recursive formula for $\chi _{j,r,s}$, which is provided in the Supplementary Information. In order to implement this QW to simulate fermions in the RSM, appropriate conditions have to be set to comply with the boundary conditions (20) and (28). It can be explicitly shown, from the recursive formula for $\chi _{j,r,s}$, that this QW dynamics respects (28), in the sense that, if the walker obeys the condition

42 $$\begin{aligned} \chi _{j,r,-s}=\eta \sigma _{z}\chi _{j,r,s}, \end{aligned}$$

at time *j*, it is also obeyed at time $j+1$. For the simulations, we discretize the *y* coordinate along the segment $[-L,L]$ with a spacing $\varepsilon$, and impose an initial condition which satisfies Eq. (42). We use the same lattice spacing in the *x* direction, together with an strategy that adapts its effective extension to the time step. We also impose periodic boundary conditions on the grid to respect condition (20), taking into account that functions evaluated at $y=L+\varepsilon$ should be identified with functions at $y=-L+\varepsilon$ to respect the periodicity in the range $[-L,L]$.

As discussed above, the parameter that governs the amount of warp in the extra dimension is given by the product *kL*. One can wonder how the ordinary spacetime limit (corresponding, in our case, to just one spatial dimension *x*) can be recovered. To this end, we consider two different lattice spacing $\varepsilon _{x}$ and $\varepsilon _{y}$ along the *x* and *y* directions, respectively. We first impose the limit $kL\rightarrow 0$, so that the vierbein becomes trivial (or, in other words, the metric $g_{\mu \nu }$ becomes the Minkowski metric). Still, *U* will contain the displacement operator $S_{y}(-\varepsilon _{y}/2)$ along a hidden closed dimension *y*. To get rid of it, we just need to further take the limit $\varepsilon _{y}\rightarrow 0$, which yields

43 $$\begin{aligned} U\rightarrow S_{x}(-\varepsilon _{x}). \end{aligned}$$

The above unitary operator can be interpreted as a QW which describes, in the continuum limit, the one-dimensional Dirac equation of a massless particle, as a special case of the model.

## Results

The QW defined in the previous section is guaranteed to reproduce (in the continuum limit) a Dirac equation of the form (16), such as the one corresponding to the RSM. The question that arises concerns the dynamics appearing at a finite lattice and time step spacing. Of course, one does not expect the QW to behave exactly as the continuum field but, to what extent do they differ? Are there any new features that appear in the discrete case? In particular, we are interested in looking for some kind of probability concentration towards the visible brane, for a given initial condition. In this Section we explore all these features.

### Stationarity of the eigenstates solutions on the quantum walk

**Figure 3:**
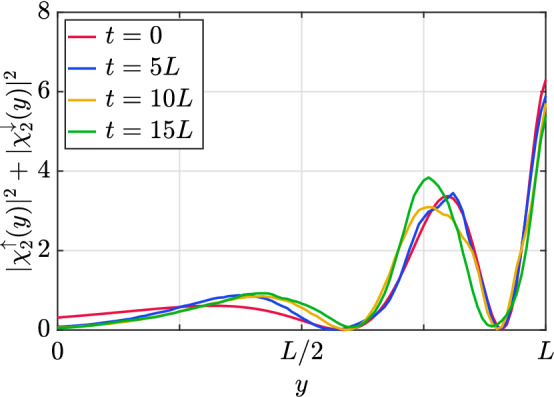
Snapshots of the probability density starting from an initial eigenstate with $n=2$ and positive energy, for a value of $kL=3$, and $q=10$. The simulation grid has 100 points along the *y* direction, and enough points have been taken in the *x* direction to ensure that the total probability density does not leak outside the boundaries.

As an initial comparison, we start by considering the discretized version of the eigenstates corresponding to the continuum limit Hamiltonian, obtained before. Such states remain stationary within this limit (i.e. they just evolve by adopting a trivial phase). How do they evolve under the action of the QW? We consider an initial state which corresponds to an eigenstate of the continuum, with fixed momentum *q*, and check whether the QW evolution of this state is stationary. The initial condition of the walker is therefore

44 $$\begin{aligned} \chi _{0,r,s}={\tilde{\phi }}_{n}(q,\varepsilon s)e^{iq\varepsilon r}, \end{aligned}$$

which represents a constant probability density along the ordinary dimension *x*. As expected, the QW evolution does not remain stationary, although it keeps a close resemblance to the initial state. This can be observed from Fig. 3, where we represented the normalized marginal probability along the *y* direction of the walker (after summing over *x*) at different time steps, for an initial stationary state solution with $n=2$, and warp coefficient $kL=3$.

### Localization in the QW

**Figure 4:**
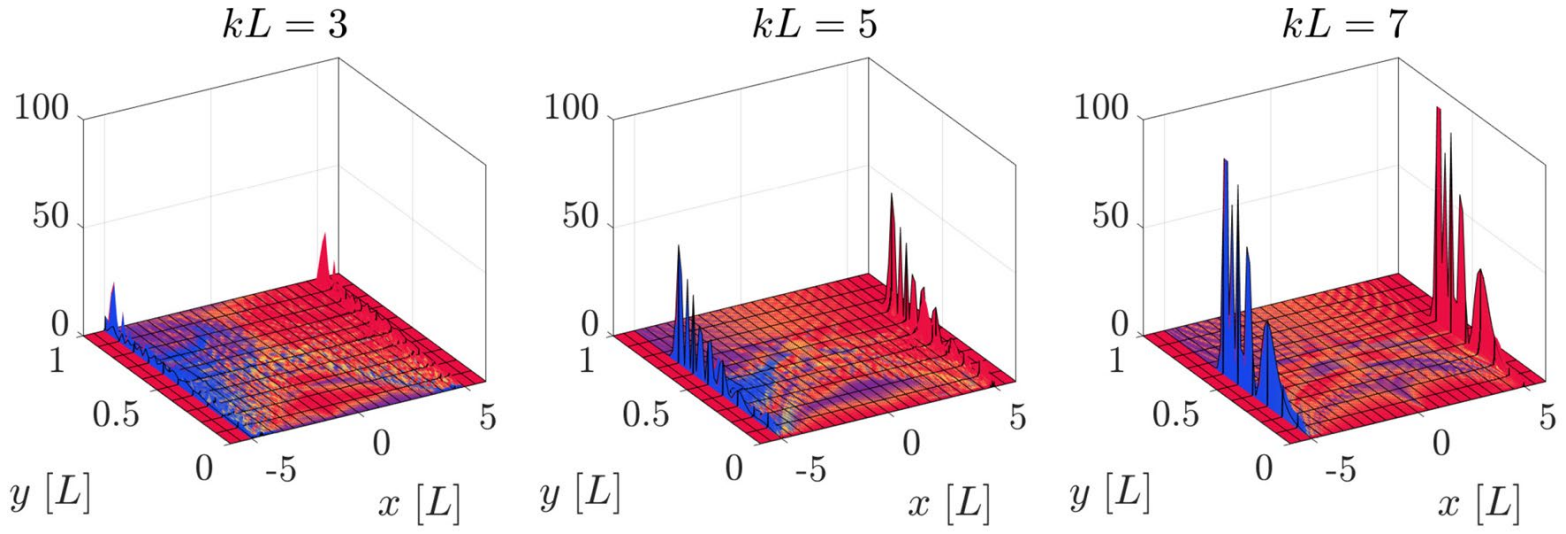
Probability density distribution, at $t=5L$, of an initial localized walker centred at $(x_{0},y_{0})=(0,L/2)$ for different values of *kL* with initial coin components $C_{0}=\frac{1}{\sqrt{2}}(1,i)^{T}$. The height of the curve represents the probability of finding the walker in that position, and the colors indicate the coin state. The red (blue) color indicates a predominance of the upper (lower) component, while yellow stands for a superposition of both components. The simulation grid has 100 points along the *y* direction, and enough points have been taken in the *x* direction to ensure that the total probability density does not leak outside the boundaries.

We now investigate the localization capability of the above defined QW, i.e., whether it shows a tendency to concentrate the walker towards the visible brane at $y=L$. We consider an initial walker which is fully localized

45 $$\begin{aligned} \chi _{0,r,s}^ {}=\delta _{x,0}\delta _{y,y_{0}}C_{0}, \end{aligned}$$

where $C_{0}$ is the initial coin state, and we recall that $x=\varepsilon r$ and $y=\varepsilon s$. We explore the evolution of a walker which is initially localized at the center of the extra dimension, that is at $y_{0}=\frac{L}{2}$, and we study the probability distribution for different values of the warp coefficient, at a given time step. In Fig. 4 we show the surface plot of the probability density with the above initial conditions, and $C_{0}=\frac{1}{\sqrt{2}}(1,i)^{T}$, which induces a symmetric evolution in the ordinary dimension. The blue (red) color of the surface represents dominance of the upper (lower) coin component, while yellow stands for a superposition of both components.

**Figure 5:**
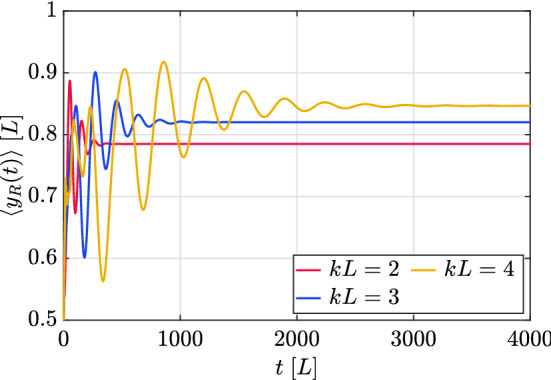
Expected value of the probability distribution along the extra dimension *y*, as calculated from the FPD, for different values of the warp coefficient *kL*. The initial condition is the same as in Fig. 4.

We notice that most of the probability distribution in the *x* direction is concentrated along a freely propagating front which moves at the maximum speed ($x=\pm t$), consistently with the fact that the QW simulates massless fermions. We also notice that most of the right propagating distribution (positive values of *x*) is dominated by the upper coin component, while the part propagating to the left (negative values of *x*) mainly contains the lower coin component, a fact that can also be inferred from the explicit evolution of the QW (see Supplementary Information for details). The propagation of the walker along the extra dimension *y* strongly depends on the value of the warp coefficient. At $t=5L$, the distribution with the lowest value of *kL* possesses non-zero values on the visible brane $y=L$, while the other two do not. In fact, the displacement of the probability distribution towards $y=L$ is slower for the highest *kL*. In other words, a larger value of the warp coefficient dramatically increases the time scale of the dynamics along the extra dimension, and makes it prohibitively expensive (in terms of computational cost) to explore larger values of *kL* than those considered here.

In order to investigate whether the QW exhibits the same behavior as the stationary states, in the sense that a higher value of the warp coefficient induces a stronger localization near the visible brane, we study the distribution of the freely propagated parts of the walker (the regions around $x=\pm t$), where most of the probability density is concentrated, as can be readily seen in Fig. 4. The probability distribution associated to these two zones will be referred to as the “freely propagating distribution” (FPD). In terms of the spinor components, those are the probability density distributions obtained from $\chi _{j,s}^{\mathrm {R}}\equiv \chi _{j,j,s}$ and $\chi _{j,s}^{\mathrm {L}}\equiv \chi _{j,-j,s}$, where $r=\pm j$ restricts the wavefunction to the two freely propagating peaks. In Fig. 5 we represent the expected value for these distributions along the *y* dimension, which can be defined as

46 $$\begin{aligned} \langle y_{R(L)}(t)\rangle =\sum _{s}\varepsilon s\;\chi _{j,s }^{\mathrm {R(L)}\dagger }\chi _{j,s}^{\mathrm {R(L)}}, \end{aligned}$$

where $t=\varepsilon j$, for different values of *kL*. First of all we notice that this quantity reaches an asymptotic value, which is closer to *L* for higher warp coefficients. Secondly, as discussed above, the warp coefficient induces a change in the time scale of the dynamics, so that lower values of the warp coefficient show a faster convergence towards the asymptotic state, consistently with the features already observed in Fig. 4.

**Figure 6:**
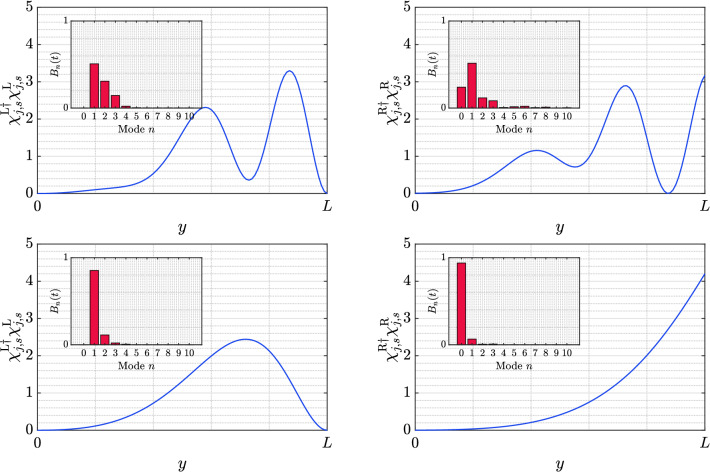
Probability distributions of the FPDs along the extra dimension *y*, for the value $kL=3$. The inset is a histogram showing the value of the $B_{n}(t)$ coefficients, as defined by Eq. (48): see the text for an explanation. The left (right) panels show the left (right) FPD. The top panels are calculated at a shorter time $t=50L$ and the bottom ones at a longer time $t=1000L$. The initial condition is the same as in Fig. 5, and the simulation grid has 200 points along the *y* direction.

### Mode decomposition of the freely propagating distribution

Our simulations indicate that the FPD reaches a steady state along the extra dimension, in a similar fashion as the expected value (46). This evolution can be appreciated from the plots of Fig. 6. Al late times (lower row), the probability distribution resembles the probability density of a stationary state with positive energy and momentum in one of the lowest modes: $n=0$ for the right FPD, and $n=1$ for the left FPD. It is important to recall that, as discussed above, the right (left) FPD is predominantly composed by the upper (lower) component of the spinor, and that $n=0$ has no lower component: see Eq. (36). This causes a fundamental difference when comparing the left and right contributions. In order to investigate these features on the time evolution, we introduce a decomposition on the wavefunction of the walker as a combination of the stationary states basis. This allows us to write

47 $$\begin{aligned} \chi _{j,r,s}=\int _{-\pi /\varepsilon }^{\pi /\varepsilon }\frac{dq}{2\pi } \sum _{n}\beta _{n}(q,t){\tilde{\phi }}_{n}(q,\varepsilon s)e^{-iq\varepsilon r}, \end{aligned}$$

where the temporal dependence is included on the $\beta _{n}(q,t)$ coefficients. In the Supplementary Information we detail how these factors can be computed, and define their normalization conditions. In particular, we are interested on the contribution of each value *n*, therefore we integrate out the dependence in the quasi-momentum *q*. In other words, we are interested on the following (time-dependent) coefficients:

48 $$\begin{aligned} B_{n}(t)=\int _{-\pi /\varepsilon }^{\pi /\varepsilon } \frac{dq}{2\pi }\left| \beta _{n}(q,t)\right| ^{2}. \end{aligned}$$

The different mode components $B_{n}(t)$ of Fig. 6 have been included as an inset in those plots. On the one hand, it can be observed that, at long times, when a steady state has been reached, the FPDs are mostly composed by the lowest possible mode ($n=0$ or $n=1$, as discussed above). On the other hand, at short times, the FPDs contain additional higher modes.

### Entanglement entropy

Finally, we study the entanglement properties that the QW exhibits between the coin and position degrees of freedom for the already considered, initially localized state. The entanglement can be quantified using the von Neumann entropy of the reduced density matrix in the coin space

49 $$\begin{aligned} S(t)=-\mathrm {Tr}\left\{ \rho _{c}(t)\log _{2}\rho _{c}(t)\right\} , \end{aligned}$$

where $\rho _{c}(t=\varepsilon j)=\sum _{r,s}\chi _{j,r,s}\chi _{j,r,s}^{\dagger }$ is the reduced density matrix in the coin space, i.e. after tracing out the spatial degrees of freedom. In the simple case of a QW on a line with a constant coin operator, the entanglement primarily arises as a consequence of the presence of the spin-dependent displacement operator *S* in the unitary *U*, although it can be modulated by both the angle of the coin operator and by the initial state^32,33^. For the same reason, we also expect entanglement to be produced in our model, although an analytical calculation, similar to previous references, is probably unfeasible for a 2D spatial case which, moreover, includes position-dependent unitaries, as in Eq. (38).

**Figure 7:**
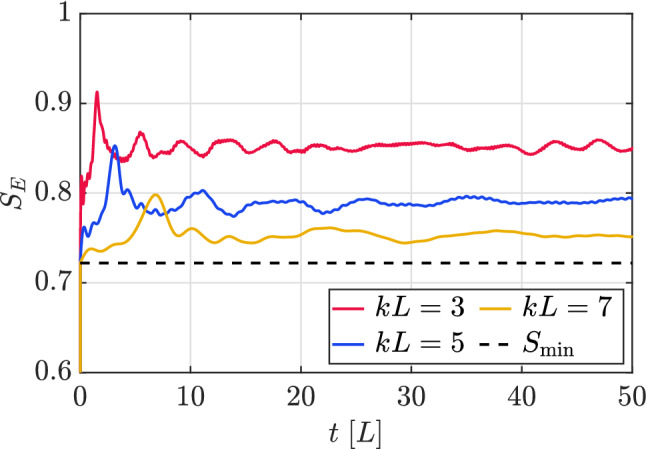
Evolution of the entanglement entropy with the initial condition Eq. (45) centred at $y_{0}=L/2$ for different values of the warp coefficient and initial coin components $C_{0}=\frac{1}{\sqrt{5}}(1,2i)$. The dotted line represent the minimum value the entropy can reach for very high values of *kL*, which is computed in the Supplementary Information. This simulation grid has 50 points along the *y* direction.

**Figure 8:**
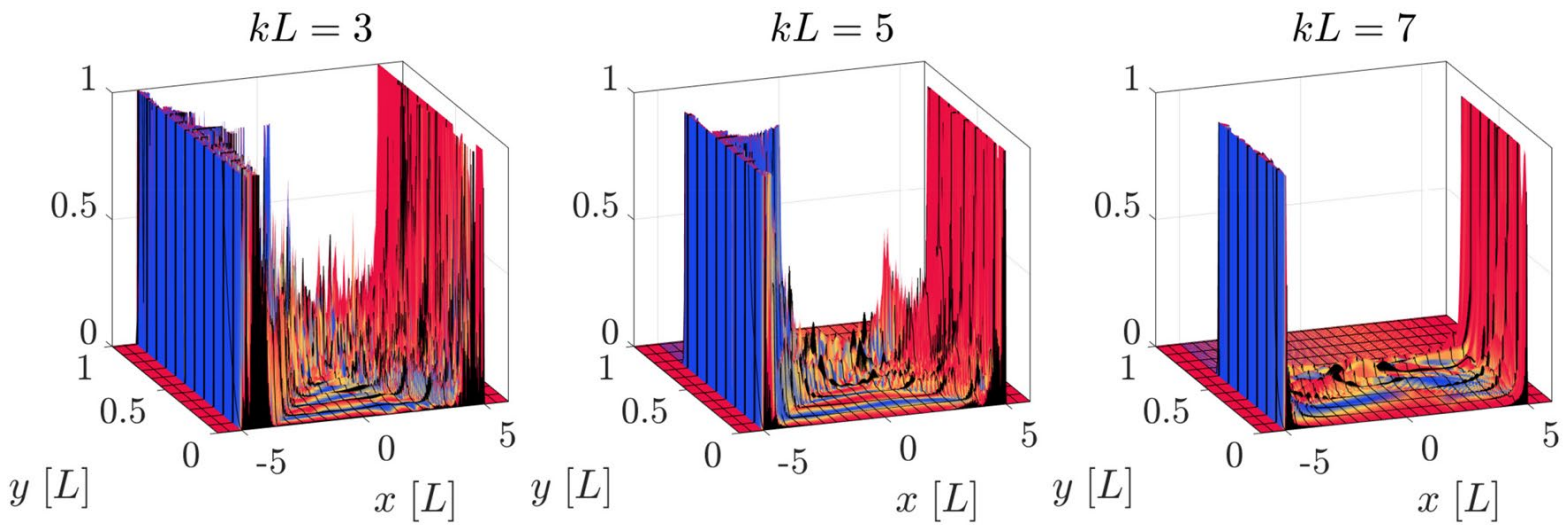
Probability density distribution, at $t=5L$, for an initially localized walker centered at $(x_{0},y_{0})=(0,L/2)$, and different values of *kL*, with initial coin components $C_{0}=\frac{1}{\sqrt{5}}(1,2i)^{T}$. The vertical axis has been zoomed in to show that the probability density between the two regions of the FPD is more scrambled for lower values of the warp coefficient. The colors and grid parameters are the same as in Fig. 4.

In Fig. 7 we plot the evolution of the entanglement entropy of a fully localized initial state for different values of the warp coefficient, with a coin state $C_{0}=\frac{1}{\sqrt{5}}(1,2i)^{T}$. Notice that this choice is different from that one used in the previous section, for reasons that are explained below. It can be seen that the entanglement entropy reaches lower values as *kL* increases, an effect that can probably be due to the fact that the probability density in between the FPDs becomes more spread (and therefore “less ordered”) at lower values of *kL*. This can be observed in Fig. 8, where we plotted a zoomed version of Fig. 4, but obtained with the above initial coin components $C_{0}=\frac{1}{\sqrt{5}}(1,2i)^{T}$. One can see that, for lower values of the warp coefficient, a significant part of the probability distribution is scrambled in the intermediate region between both parts of the FPD. This diffusion effect can be totally mitigated for extreme values of the warp coefficient, leading to a minimum value of the entropy which is completely dominated by the FPD, and can be obtained from the initial coin components. In the Supplementary Material we show this limiting situation, and how the corresponding entropy can be computed. The initial coin state $C_{0}=\frac{1}{\sqrt{2}}(1,i)^{T}$ previously used produces values of the entropy which are very close to unity in all cases, making it difficult to appreciate the effects that are discussed above.

## Conclusions

We have investigated a quantum walk which allows to simulate the Randall–Sundrum model of extra dimensions, while satisfying the constrains imposed by the symmetries of that model. This model has played an important role in high energy physics, aiming to solve the hierarchy problem, by introducing one finite extra dimension that possesses two branes at its extremes. The matter fields are confined in the visible brane, while gravity is allowed to span along this whole dimension. We worked it out for the case of spin 1/2 fermions in a two dimensional space, composed by an ordinary dimension and an orbifolded one, apart from time, and obtained the Dirac equation in this spacetime configuration. The boundary conditions of the orbifold on the fermionic field forced it to be massless on the bulk. In this lower dimensional space we were able to obtain the eigenenergies of the fermionic field, as well as the corresponding eigenstates, showing a probability density which is concentrated near the visible brane, a phenomenon that bears an analogy with the localization effect that can be found in many scenarios^19–21,23,24,26,27^.

This analogy motivated us to seek localization effects on the QW that we introduced to simulate the RSM. The QW is defined in such a way that, in the continuum limit, the Dirac equation of the fermionic field for the RSM metric is recovered. We investigated the confining capabilities of the QW, by considering an initially localized walker away from the visible brane. We concluded that the freely propagating parts of the probability distribution, where the probability is mostly concentrated, reach an asymptotic value of the expected position along the extra dimension. Moreover, the asymptotic value gets closer to the visible brane for higher values of the warp coefficient, which therefore drives the strength of localization, and also noticed that it had an effect on the timescale of the dynamics, by delaying them for higher values of the coefficient.

At long time steps, the probability densities show an asymptotic shape, with a resemblance with the eigenstates that were obtained in the continuous model, which suggested a study based on the decomposition of the wavefunction in terms of these stationary states. We found that the freely propagating parts of the QW are dominated, in the asymptotic regime, by the lowest possible (i.e., compatible with the symmetries of the model) modes. At intermediate time steps, the same decomposition manifests a combination of multiple modes with higher energy.

Finally, we found that the entanglement between coin and spatial degrees of freedom is reduced for stronger warp coefficients. We associated this result to the higher spreading of the density distribution for the lower values of the warp coefficient.

We conclude that quantum walks are suitable candidates for simulating models of field theories with extra dimensions that rely on the curvature of the spacetime. Not only the model is interesting from the point of view of the field theory: It allows to design a quantum process that can be tailored to exhibit very rich dynamics, showing free propagation in one dimension, and an asymptotic confining behavior on the other one, with rates that can be tuned by an appropriate choice of the parameters. In this way, the interplay between high energy physics and quantum simulations can be of mutual benefit.

## Supplementary Information

Supplementary Information.
