# Supplementary material for: A quantum walk simulation of extra dimensions with warped geometry
Source: Sci Rep. 2022 Feb 4;12:1926. doi: 10.1038/s41598-022-05673-2 (PMC8816941; doi:10.1038/s41598-022-05673-2)
Supplement: Supplementary file 1 — Supplementary Information. [file 41598_2022_5673_MOESM1_ESM.pdf]

Supplementary Material: A quantum walk simulation of extra dimensions with warped geometry

Andreu Anglés-Castillo[1,*] **and Armando Pérez**[1]

[1]Universitat de València-CSIC, Departament de Física Teòrica & IFIC, Burjassot (València), 46100, Spain
[*]andreu.angles@ific.uv.es

Metric Solution

Here we prove that the metric (6) extremizes the background action (3), which can be rewritten as

$$S = \int dy \int dx^{\mu} \sqrt{|g|} \Big(2\alpha\mathscr{R} - \Lambda - \delta(y) T_{\text{hid}} - \delta(y-L) T_{\text{vis}} \Big) \ . \tag{S.1}$$

The extrema of this action gives the following Einstein equations

$$\sqrt{|g|}\left(R_{MN} - \frac{1}{2} g_{MN}\mathscr{R} + \frac{1}{4\alpha}\Lambda g_{MN} \right) = -\frac{\sqrt{|g|}}{4\alpha}\left(T_{\text{hid}}\delta(y) g_{\mu\nu}\delta^{\mu}_{M}\delta^{\nu}_{N} + T_{\text{hid}}\delta(y-L) g_{\mu\nu}\delta^{\mu}_{M}\delta^{\nu}_{N} \right) , \tag{S.2}$$

with indices $M,N = \{t,x,y\}$, while $\mu,\nu = \{t,x\}$ only account for ordinary dimensions. After computing the curvature tensor, we obtain the following equation for the yy component

$$A'(y)^2 + \frac{\Lambda}{4\alpha} = 0 \ , \tag{S.3}$$

which yields the solution

$$A(y) = k|y| \ , \quad \text{with} \quad k \equiv \sqrt{-\frac{\Lambda}{4\alpha}} \ , \tag{S.4}$$

and has to be consistent with the orbifold symmetry (2). There are no μy components, as the metric and tensors with these components vanish. The $\mu\nu$ components of the Einstein equations are

$$(A'(y)^2 - A''(y)) e^{-2A(y)}\eta_{\mu\nu} + \frac{1}{4\alpha}\Lambda e^{-2A(y)}\eta_{\mu\nu} = -\frac{1}{4\alpha} e^{-2A(y)}\eta_{\mu\nu}\left[T_{\text{hid}}\delta(y) + T_{\text{vis}}\delta(y-L) \right] \ , \tag{S.5}$$

which, making use of equation (S.3), can be simplified to

$$A''(y) = \frac{1}{4\alpha}\left[T_{\text{hid}}\delta(y) + T_{\text{vis}}\delta(y-L) \right] \ . \tag{S.6}$$

After computing the second derivative of $A(y)$ from Eq. (S.4), and taking into account the periodicity of the metric (1), yields

$$A''(y) = 2k(\delta(y) - \delta(y-L)) \ , \tag{S.7}$$

which allows us to identify, from Eq. (S.6), the values of the tensions

$$-T_{\text{vis}} = T_{\text{hid}} = 8\alpha k = \sqrt{-16\Lambda\alpha} \ . \tag{S.8}$$

The results obtained in this section indicate that the bulk geometry has to be Anti-de Sitter, with a negative bulk cosmological constant, and that the visible brane has negative tension, while the hidden one is positive. These results differ, from standard works on Randall-Sundrum, on the constant coefficient appearing in the expression for k, Eq. (S.4) because we are considering a one dimensional ordinary space, so that the computations of the curvature tensor yield different constant factors.

Hamiltonian eigenstates

In order to solve the eigenvalue problem (30), it is convenient to perform the change of basis $\xi(q,y) = H\tilde{\phi}(q,y)$, with

$$H = \begin{pmatrix} 1 & 1 \\ 1 & -1 \end{pmatrix} \tag{S.9}$$

the Hadamard matrix, so that the eigenvalue equation becomes

$$\left[q + \frac{i}{2}\left(p_y e^{-A(y)} + e^{-A(y)} p_y\right)\right] \xi_n^- = E_n \xi_n^+ \,, \tag{S.10}$$

$$\left[q - \frac{i}{2}\left(p_y e^{-A(y)} + e^{-A(y)} p_y\right)\right] \xi_n^+ = E_n \xi_n^- \,, \tag{S.11}$$

where $\xi^\pm$ are the components of $\xi = (\xi^+, \xi^-)^T$. This system of equations can be decoupled, giving

$$\left[q^2 + \frac{1}{4}\left(p_y e^{-A(y)} + e^{-A(y)} p_y\right)^2\right] \xi_n^\pm = E_n^2 \xi_n^\pm \,, \tag{S.12}$$

which is a second order differential equation that can be solved for the appropriate boundary conditions. We solve this equation both in the positive $[\xi^\pm(0<y<L)]_P$ and negative domain $[\xi^\pm(-L<y<0)]_N$, delivering

$$\left[\xi_n^\pm(y)\right]_P = A e^{\frac{ky}{2}} \cos\left(e^{ky}\alpha_n\right) + B e^{\frac{ky}{2}} \sin\left(e^{ky}\alpha_n\right) \,, \tag{S.13}$$

$$\left[\xi_n^\pm(y)\right]_N = C e^{-\frac{ky}{2}} \cos\left(e^{-ky}\alpha_n\right) + D e^{\frac{ky}{2}} \sin\left(e^{-ky}\alpha_n\right) \,, \tag{S.14}$$

where we defined

$$\alpha_n = \frac{\sqrt{E_n^2 - q^2}}{k} \,. \tag{S.15}$$

These solutions are related by the continuity conditions

$$\left[\xi_n^\pm(0)\right]_P = \left[\xi_n^\pm(0)\right]_N \,, \text{ and } \left[\xi_n^\pm(L)\right]_P = \left[\xi_n^\pm(-L)\right]_N \,, \tag{S.16}$$

where in the last one the periodicity of the wavefunctions, Eq. (20), has been used, and imply that the solutions are related by $A = C$ and $B = D$. The discontinuity introduced by the delta terms at $y = 0$ and $y = \pm L$, coming from $A''(y)$, imposes $B = A\tan\alpha_n$, and the following restrictions to the energies

$$\tan\alpha_n = \tan\left(e^{kL}\alpha_n\right) \,, \tag{S.17}$$

which yields the spectrum in Eq. (32). After taking into account these conditions, the eigenstates become

$$\xi_n^\pm(y) = A e^{\frac{k|y|}{2}} \left[\cos\left(e^{k|y|}\alpha_n\right) + \tan\alpha_n \sin\left(e^{k|y|}\alpha_n\right)\right] \,. \tag{S.18}$$

However, these solutions come from the second order differential equation (S.12), whereas the original equations were first order, and relate $\xi^+(y)$ to $\xi^-(y)$. To find the appropriate solution of the eigenfunctions, we need to take into account these relations. Since any lineal combination of solutions is also a solution of the equations, we consider the solution $[\xi_n^+(y)]_2$ of Eq. (S.12) to obtain $[\xi_n^-(y)]_1$ from Eq. (S.11), where $[\,\cdot\,]_i$ denotes whether the solution comes from a first ($i=1$) or second ($i=2$) order differential equation. Similarly, from $[\xi_n^-(y)]_2$ we obtain $[\xi_n^+(y)]_1$, so that

$$\left[\xi_n^\pm(y)\right]_1 = \left[\sin\left(\alpha_n e^{k|y|}\right)\left(\frac{q}{E}\tan\alpha_n \pm \frac{k\alpha_n}{E}\mathrm{sign}(y)\right) + \cos\left(\alpha_n e^{k|y|}\right)\left(\frac{q}{E} \mp \frac{k\alpha_n}{E}\tan\alpha_n \mathrm{sign}(y)\right)\right] A e^{\frac{k|y|}{2}} \,. \tag{S.19}$$

The general solution for the eigenstates is a lineal combination of this pair of solutions

$$\xi_n^+(y) = K_1\left[\xi_n^+(y)\right]_2 + K_2\left[\xi_n^+(y)\right]_1 \,, \tag{S.20}$$

$$\xi_n^-(y) = K_1\left[\xi_n^-(y)\right]_1 + K_2\left[\xi_n^-(y)\right]_2 \,, \tag{S.21}$$

where the relation between the constants K_1 and K_2 is set by Eq. (28), which, depending on the possible values of η, implies the restrictions

$$\eta = +1 \implies K_1 = K_2 \ , \tag{S.22}$$
$$\eta = -1 \implies K_1 = -K_2 \ . \tag{S.23}$$

Finally, undoing the change of basis, we recover the original eigenstate components of Eqs. (34,35) for $\eta = +1$, while

$$\phi_n^{\uparrow}(y) = \sqrt{\frac{2k}{e^{kL}-1}} \frac{k\alpha_n}{\sqrt{(E_n+q)^2+(k\alpha_n)^2}} e^{\frac{k|y|}{2}} \sin\left[\alpha_n\left(1-e^{k|y|}\right)\right] \operatorname{sign}(y) \ , \tag{S.24}$$
$$\phi_n^{\downarrow}(y) = \sqrt{\frac{2k}{e^{kL}-1}} \frac{E_n+q}{\sqrt{(E_n+q)^2+(k\alpha_n)^2}} e^{\frac{k|y|}{2}} \cos\left[\alpha_n\left(1-e^{k|y|}\right)\right] \ , \tag{S.25}$$

are obtained for $\eta = -1$, and where the remaining constant was set by the normalization of the wavefunction

$$\int_0^L dy \tilde{\phi}_n(q,y)^\dagger \tilde{\phi}_n(q,y) = 1 \ . \tag{S.26}$$

The solution for the particular case of $n = 0$ has only a lower component, and is given by

$$\phi_0^{\downarrow}(y) = \sqrt{\frac{k}{e^{kL}-1}} e^{\frac{k|y|}{2}} \operatorname{sign}(E_n+q) \ . \tag{S.27}$$

QW explicit time step

In[9] the following QW operator was proposed as a way to reproduce the continuous limit of the Dirac equation in a curved space time with 2 spatial dimensions

$$U = \Pi^{-1}\left[W_x\left(\theta^{12}\right)W_y\left(\theta^{22}\right)\right]\Pi\left[W_y\left(\theta^{21}\right)W_x\left(\theta^{11}\right)\right] \ , \tag{S.28}$$

where the angles $\{\theta^{ab} \ / a,b = 1,2\}$ are allowed to depend, in general, both on the time index j and the spatial coordinates x,y. In our case, the metric does not depend on time, therefore we have dropped the subindex j appearing in the above reference. We have also omitted a factor which depends on the mass, since we are interested in the massless case. The matrix Π reads

$$\Pi = \frac{1}{\sqrt{2}}\begin{pmatrix} -i & 1 \\ -1 & i \end{pmatrix}. \tag{S.29}$$

For a given θ, the operator $W_k(\theta)$, for each $k = x,y$, is defined by

$$W_k(\theta) = r^{-1}(\theta)\, u(\theta)\, S_k(-\varepsilon/2) u(\theta)\, S_k(-\varepsilon/2) r(\theta) \ , \tag{S.30}$$

with matrices

$$r(\theta) = \begin{pmatrix} i\cos\theta/2 & i\sin\theta/2 \\ -\sin\theta/2 & \cos\theta/2 \end{pmatrix} \ , \quad u(\theta) = \begin{pmatrix} -\cos\theta & i\sin\theta \\ -i\sin\theta & \cos\theta \end{pmatrix} \ , \tag{S.31}$$

and $S_k(\varepsilon) = \exp(-i\varepsilon p_k \sigma_z)$ the spin-dependent shift operator along the k direction. We have checked that, in the continuum limit, this QW reproduces the Dirac equation (16) (for $m = 0$), with B^x and B^y given by

$$B^x = \begin{pmatrix} -\cos\theta^{11} & -i\cos\theta^{12} \\ i\cos\theta^{12} & \cos\theta^{11} \end{pmatrix}, \tag{S.32}$$

$$B^y = \begin{pmatrix} -\cos\theta^{21} & -i\cos\theta^{22} \\ i\cos\theta^{22} & \cos\theta^{21} \end{pmatrix}. \tag{S.33}$$

A direct comparison with Eq. (18) provides the identification

$$\cos\theta^{11} = 1 \ , \tag{S.34}$$
$$\cos\theta^{12} = 0 \ , \tag{S.35}$$
$$\cos\theta^{21} = 0 \ , \tag{S.36}$$
$$\cos\theta^{22} = e^{-A(y)} \ , \tag{S.37}$$

which allows us to simplify the operators $W_k(\theta)$, resulting in $W_x(\theta^{12}) = W_y(\theta^{21}) = \mathbb{I}$, and $W_x(\theta^{11}) = S_x(-\varepsilon)$. With these simplifications we finally obtain Eq. (38).

Making use of the equations that define the QW, Eqs. (37, 38, 39) and (40), one can recast the evolution of $|\chi_j\rangle$ as a recurrence relation relating the spinor components Eq. (41) at two consecutive time steps. We arrive at

$$\begin{aligned}\chi^{\uparrow}_{j+1,r,s} =& -\frac{i}{2}e^{i\theta(y)}\left[s\left(y+\frac{\varepsilon}{2}\right)+s\left(y-\frac{\varepsilon}{2}\right)\right]\chi^{\uparrow}_{j,r+1,s} - \frac{1}{2}\left[s\left(y+\frac{\varepsilon}{2}\right)-s\left(y-\frac{\varepsilon}{2}\right)\right]\chi^{\downarrow}_{j,r-1,s}\\ &+\frac{1}{2}f(y)f(y+\varepsilon)c\left(y+\frac{\varepsilon}{2}\right)\chi^{\uparrow}_{j,r+1,s+1} + \frac{1}{2}f(y)f(y-\varepsilon)c\left(y-\frac{\varepsilon}{2}\right)\chi^{\uparrow}_{j,r+1,s-1}\\ &+\frac{i}{2}f(y)f^*(y+\varepsilon)c\left(y+\frac{\varepsilon}{2}\right)\chi^{\downarrow}_{j,r-1,s+1} - \frac{i}{2}f(y)f^*(y-\varepsilon)c\left(y-\frac{\varepsilon}{2}\right)\chi^{\downarrow}_{j,r-1,s-1}\ ,\end{aligned} \tag{S.38}$$

for the upper component, where we recall that $y = \varepsilon s$, and we defined $e^{\pm i\theta(y)} = c(y) \pm is(y)$. For the lower component one finds

$$\begin{aligned}\chi^{\downarrow}_{j+1,r,s} =& \frac{i}{2}e^{-i\theta(y)}\left[s\left(y+\frac{\varepsilon}{2}\right)+s\left(y-\frac{\varepsilon}{2}\right)\right]\chi^{\downarrow}_{j,r-1,s} - \frac{1}{2}\left[s\left(y+\frac{\varepsilon}{2}\right)-s\left(y-\frac{\varepsilon}{2}\right)\right]\chi^{\uparrow}_{j,r+1,s}\\ &+\frac{1}{2}f^*(y)f^*(y+\varepsilon)c\left(y+\frac{\varepsilon}{2}\right)\chi^{\downarrow}_{j,r-1,s+1} + \frac{1}{2}f^*(y)f^*(y-\varepsilon)c\left(y-\frac{\varepsilon}{2}\right)\chi^{\downarrow}_{j,r-1,s-1}\\ &-\frac{i}{2}f^*(y)f(y+\varepsilon)c\left(y+\frac{\varepsilon}{2}\right)\chi^{\uparrow}_{j,r+1,s+1} + \frac{i}{2}f^*(y)f(y-\varepsilon)c\left(y-\frac{\varepsilon}{2}\right)\chi^{\uparrow}_{j,r+1,s-1}\ .\end{aligned} \tag{S.39}$$

We notice that the upper components are displaced in one direction along the x dimension, while the lower components are displaced in the opposite direction.

Mode decomposition of the freely propagating distribution

The stationary states found above form an orthonormal basis, in the continuum limit, that allow for a decomposition of any function along the y coordinate, for a given value of q. They can also be used, after a proprer discretization, in the lattice on which the QW is defined. Following this idea, we introduced the decomposition in Eq. (47), which is a function in the space of q, the lattice quasimomentum along the x coordinate. For this quasimomentum space, the spinor components are related to Eq. (41) via a discrete Fourier transform

$$\tilde{\chi}_{j,s}(q) = \sum_r e^{-iq\varepsilon r}\chi_{j,r,s}\ . \tag{S.40}$$

Making use of

$$\sum_r e^{ix(q-q')} = \frac{2\pi}{\varepsilon}\delta(q-q')\ , \tag{S.41}$$

and the orthonormality condition (S.26) on the grid

$$\varepsilon\sum_s \tilde{\phi}_n(q,\varepsilon s)^{\dagger}\tilde{\phi}_m(q,\varepsilon s) = \delta_{n,m}\ , \tag{S.42}$$

the coefficients can be obtained as

$$\beta_n(q,t) = \varepsilon^2\sum_s \tilde{\phi}_n(q,\varepsilon s)\tilde{\chi}_{j,s}(q)\ . \tag{S.43}$$

The coefficients of the freely propagating distribution with $x = t$ are

$$\beta_n(q,t) = \varepsilon^2\sum_s \tilde{\phi}_n(q,\varepsilon s)e^{-iqt}\chi_{j,j,s}\ , \tag{S.44}$$

while, for $x = -t$, they read as

$$\beta_n(q,t) = \varepsilon^2\sum_s \tilde{\phi}_n(q,\varepsilon s)e^{iqt}\chi_{j,-j,s}\ . \tag{S.45}$$

From the normalization condition of the spinor on the grid

$$\varepsilon^2\sum_{r,s}\chi^{\dagger}_{j,r,s}\chi_{j,r,s} = 1\ , \tag{S.46}$$

and making use of the definition (47), it can be shown that the mode coefficients satisfy

$$\sum_n \int_{-\pi/\varepsilon}^{\pi/\varepsilon} \frac{dq}{2\pi} |\beta_n(q,t)|^2 = 1 \ , \tag{S.47}$$

which can be expressed in terms of the integrated coefficients (48) as

$$\sum_n B_n(t) = 1 \ . \tag{S.48}$$

High kL limit of the QW time step and limiting entropy

In the limit of a high warp factor kL, the exponential $e^{-A(L)}$ becomes very small, so that the QW discrete time recursive evolution Eqs. (S.38,S.39) can be expanded up to the lowest order in this factor, giving

$$\begin{aligned} \chi^{\uparrow}_{j+1,r,s} =& \chi^{\uparrow}_{j,r+1,s} \ , \\ \chi^{\downarrow}_{j+1,r,s} =& \chi^{\downarrow}_{j,r-1,s} \ . \end{aligned} \tag{S.49}$$

Although this expansion is only valid for values of y close to L, it is still accurate enough for the initial condition located at $y = L/2$. As discussed in the main text, the asymptotic value of the entanglement entropy decreases as kL is increased. Therefore, the minimum value of the entropy is reached in the limit $e^{-A(L)} \approx 0$. The initial condition $\chi_{0,r,s} = \delta_{r,0}\delta_{s,s_0}C_0$ can be iterated with the help of Eqs. (S.49) to produce the explicit time evolution

$$\begin{aligned} \chi^{\uparrow}_{j,r,s} =& C^{\uparrow}_0 \delta_{r,j} \delta_{s,s_0} \ , \\ \chi^{\downarrow}_{j,r,s} =& C^{\downarrow}_0 \delta_{r,-j} \delta_{s,s_0} \ . \end{aligned} \tag{S.50}$$

The corresponding reduced density matrix becomes time-independent and diagonal:

$$\rho_c(t) = diag(|C^{\uparrow}_0|^2, |C^{\downarrow}_0|^2) \tag{S.51}$$

from which the minimum value of the entropy can finally be obtained:

$$S_{\min} = -|C^{\uparrow}_0|^2 \log_2 |C^{\uparrow}_0|^2 - |C^{\downarrow}_0|^2 \log_2 |C^{\downarrow}_0|^2 \ . \tag{S.52}$$
